# Parents’ Relative Socioeconomic Status and Paternal Involvement in Chinese Families: The Mediating Role of Coparenting

**DOI:** 10.3389/fpsyg.2016.00940

**Published:** 2016-06-22

**Authors:** Chang Liu, Xinchun Wu, Shengqi Zou

**Affiliations:** ^1^Beijing Normal University, BeijingChina; ^2^Beijing Institute of EducationBeijing, China

**Keywords:** socioeconomic status, coparenting, paternal involvement, occupational differences/similarities, relative resource

## Abstract

This study examined the mediating role of coparenting in the association between differences/similarities in paternal and maternal socioeconomic status (SES) and paternal involvement in Chinese families. The sample included 244 couples with children aged 3–7 years. Fathers and mothers reported their individual incomes, educational levels, occupations, and coparenting behavior (measured using the Coparenting Scale), and fathers completed the Father Involvement Questionnaire. Structural equation modeling was performed to examine the associations between SES and paternal involvement. Results suggested that SES indicator measures were outcome specific. Occupational differences/similarities were associated with paternal involvement indirectly, via fathers’ family integrity practices. Income and educational differences/similarities did not affect paternal involvement. The results suggested that the traditional Chinese view that “men are chiefly responsible for activity in society, while women are responsible for the home” has faded.

## Introduction

Research examining fathering in China remains in its infancy. Most studies have focused on EMBU, a Chinese translation that assesses memories concerning parents’ child rearing styles (Shwalb et al., 2010). Little is known about Chinese paternal involvement in activities in their children’s daily lives. The scarcity of relevant research represents a major gap in the literature. The unique contributions of paternal involvement to children’s development have been documented well in both Western and Eastern cultures (Amato and Rivera, 1999; Shwalb et al., 2010). Therefore, researchers have a strong interest in investigating factors that could facilitate paternal involvement (Schoppe-Sullivan et al., 2008). Scholars have reported a significant effect of socioeconomic factors (Roopnarine et al., 2005; McLanahan and Beck, 2010), such as paternal income (Castillo et al., 2012), paid employment (Pinto and Coltrane, 2009), educational level (Hossain and Shipman, 2009; Bronte-Tinkew et al., 2010) and maternal employment (Barnett and Baruch, 1987; Suppal and Roopnarine, 1999), on paternal involvement. Most of these studies considered paternal and maternal socioeconomic status (SES) separately, and parents’ relative SES has not received sufficient attention.

Considering SES of parents, the relative resource model (e.g., Ferree, 1990) suggests that a couple’s individual resources, including income, occupational status, and educational level, confer power, and powerful parents often wish to reduce the extent of their own household labor (Mannino and Deutsch, 2007). Considering household duties, although childcare is more of a joy than it is housework, high-earning parent seek to oﬄoad onerous parenting tasks (e.g., taking children to the doctor) onto the other (Raley et al., 2012).

Using the relative resource model, some studies have examined couples’ relative power within their households (Presser, 1994; Pinto and Coltrane, 2009), focusing mainly on factors such as unpaid housework and childrearing. Deutsch et al. (1993) examined parents’ relative socioeconomic contributions to housework and childrearing. They found that the greater the income discrepancy in favor of fathers, the less involved they were in infant care. However, few studies have specifically examined fathers’ and mothers’ relative socioeconomic contributions to paternal involvement, particularly in Chinese families. In modern China, as in most parts of the world, mothers’ work-related responsibilities have increased, which has improved maternal SES. However, the gender gap in employment and income is widening in China, and mothers generally have shorter careers, higher turnover rates, and lower job status and tend to work part-time (Shwalb et al., 2010).

It is notable that exploring the mediator in the association between SES and paternal involvement could reveal valuable information about SES indicators mechanism. The ecological model of coparenting (Feinberg, 2003) regards coparenting as a mediator of the influence of family factors on parenting practices. Coparenting refers to “an enterprise involving the coordination between adults responsible for the care and upbringing of a child” (McHale et al., 2002), reflecting the ways in which parents relate to each other (Feinberg, 2003). The current study adopted the framework proposed by McHale (1997), which focused on family integrity practices (behaviors that promote a sense of togetherness in family members), reprimand behavior (coparental disciplinary activities), conflict (overt interparental arguments), and disparagement (active disparagement of the coparent and undermining a partner’s authority or credibility). Fathers and mothers have more attempts at promoting a sense of togetherness among family members, more agreement to discipline their child, less interparental disagreement or conflict, and less disparagement of the partner that reflect high level of coparenting quality. This coparenting construct has been used widely in studies involving both Western and Chinese families (McHale et al., 2000; Karreman et al., 2008).

With respect to the association between SES and coparenting, researchers have found that parental education (Stright and Bales, 2003; Van Egeren, 2003) and income (Schoppe-Sullivan and Mangelsdorf, 2013) were associated with coparenting. To our knowledge, only one study, which was conducted by Belsky et al. (1995), has explored the effects of parental education on coparenting, and the results showed that parental educational levels significantly predicted supportive coparenting but did not affect the extent of unsupportive coparenting events. Coparenting also has been found to exert a strong effect on paternal involvement (Futris and Schoppe-Sullivan, 2007; Morrill et al., 2010; Goldberg, 2015). Most studies examining this effect used a combined estimate of fathers’ and mothers’ coparenting scores or focused on the effects of coparenting on mothers’ and fathers’ individual parenting. According to the family system (Minuchin, 1985), fathers and mothers interact with each other as familial subsystems. Maternal factors could also influence fathering. In the present study, we considered the mediating roles of fathers’ and mothers’ coparenting simultaneously, which also allowed us to control common effects and examine the unique contributions of fathers’ and mothers’ coparenting.

The aim of the present study was to explore the associations between parents’ relative SES and paternal involvement and examine the mediating role of coparenting in these associations. With respect to SES, there is general agreement that income, education, and occupational status are positively correlated (Bradley and Corwyn, 2002), but scores reflecting these SES indicators should not be combined to form a simple composite score (Conger and Donnellan, 2007). These indicators represent separate but related economic, social, and personal resources and are defined as material or financial capital, social capital, and human capital, respectively (Bradley and Corwyn, 2002). Material or financial capital reflects economic factors, human capital reflects knowledge and skills, and social capital reflects social connections and the individual’s status and power within a social network (Conger and Donnellan, 2007). In the present study, we examined these three SES indicators and their unique contributions to paternal involvement. We focused on the unique contributions of the differences/similarities between indicators of paternal and maternal SES, which were measured using parents’ individual incomes and parental educational levels and occupational status (Bradley and Corwyn, 2002). We used engagement (direct interaction with children), accessibility (availability to children), and responsibility (arrangement of resources for children; Lamb and Tamis-Lemonda, 2004) to measure paternal involvement and considered direct interaction or engagement and accessibility. Owing to the importance of paternal involvement in early childhood, we examined families with children aged 3–7 years. In China, most children begin kindergarten at the age of 3 years, providing mothers with the opportunity to undertake spells of full-time employment. Therefore, income and employment constitute representative economic resources for mothers with children older than 3 years of age.

Consistent with the view that relative earning predicts childrearing (Deutsch et al., 1993), we hypothesized that income differences/similarities would be significantly associated with paternal involvement. Previous research also has demonstrated that employment status (Barnett and Baruch, 1987) and educational levels (Marsiglio, 1991) play import roles in paternal involvement, when considering paternal and maternal SES simultaneously. Therefore, we hypothesized that occupational and educational differences/similarities would predict paternal involvement. Specifically, high differences of income, occupational and educational lead to lower paternal involvement. We also hypothesized that the direct effect of SES on paternal involvement would be mediated by fathers’ and mothers’ coparenting. Specifically, high differences of income, occupational and educational lead to lower family integrity and reprimand, higher conflict and disparagement, which further lead to lower paternal involvement.

## Materials and Methods

### Participants and Procedure

The sample was recruited from the Paternal Involvement Project sponsored by the Ministry of Education in the People’s Republic of China. The study was approved by the local ethical committee of Beijing Normal University. Written informed consent was obtained prior to data collection. Parents were asked to complete questionnaires separately during home visits. In return, families were provided with souvenirs and parenting advice materials. The original participants included 317 families; however, 73 families were excluded because of incomplete information concerning income, education, or occupation. The results of the analysis indicated that coparenting and paternal involvement did not differ significantly between families excluded from and included in the final sample, with *t* values ranging between -1.15 and 0.70. The final sample included 244 two-parent families with children aged between 3 and 7 years from mainland China. Based on China’s Development Index (National Bureau of Statistic of the People’s Republic of China, 2010), these families were collected from 26 provinces nationwide, with 35 families (14.3%) from developed area, 98 families (40.2%) from developing area, and 111 families (45.5%) from undeveloped area. Among the sample families, the average age of the children was 4.97 years (*SD* = 1.45), with 119 boys (48.8%) and 125 girls (51.2%); the fathers’ and mothers’ average ages were 33.67 years (*SD* = 4.63) and 31.57 years (*SD* = 4.63), respectively. Participants’ socioeconomic characteristics are shown in **Table 1**.

**Table 1:** Socioeconomic characteristics of participants.

	Father *N* (%)	Mother *N* (%)
**Monthly income**		
under 1,000 RMB	13 (5.3%)	48 (19.7%)
1000–1999 RMB	44 (18.0%)	70 (28.7%)
2000–2999 RMB	93 (38.1%)	76 (31.1%)
3000–3999 RMB	35 (14.3%)	24 (9.8%)
4000–4999 RMB	19 (7.8%)	9 (3.7%)
5000–5999 RMB	11 (4.5%)	3 (1.2%)
6000–6999 RMB	10 (4.1%)	7 (2.9%)
7000–7999 RMB	5 (2.0%)	2 (0.8%)
8000–8999 RMB	2 (0.8%)	2 (0.8%)
9000–9999 RMB	6 (2.5%)	1 (0.4%)
at least 10,000 RMB	6 (2.5%)	2 (0.8%)
**Highest completed education**		
Primary school or lower	17 (7.0%)	35 (14.3%)
Junior high school	73 (29.9%)	68 (27.9%)
Senior high school	50 (25.5%)	48 (19.7%)
Some college	95 (38.9%)	89 (36.5%)
Master degree or higher	9 (3.7%)	4 (1.6%)
**Occupation**		
Lower status	43 (17.6%)	76 (31.1%)
Low status	74 (30.3%)	55 (22.5%)
Medium status	40 (16.4%)	37 (15.2%)
High status	60 (24.6%)	68 (27.9%)
Higher status	27 (11.1%)	8 (3.3%)

*RMB, Renminbi.*

### Measures

#### SES

Socioeconomic status was based on income, parental educational levels, and occupational status (Bradley and Corwyn, 2002). Parents were asked to rate their individual monthly incomes using an 11-point scale ranging from 1 (*under 1,000 Renminbi*) to 11 (*at least 10,000 Renminbi*). Parents’ highest levels of completed education were measured using a five-point scale ranging from 1 (*primary school or lower*) to 5 (*master’s degree or higher*). Fathers’ and mothers’ occupations were self-reported. Occupation was coded using a five-point occupational status scale ranging from 1 (*lower status*, e.g., *without regular employment*) to 5 (*higher status*, e.g., *senior administration officials*) based on standard occupational classification (Shi and Shen, 2007), which has been used widely in coding occupational status in China.

Each indicator for fathers’ and mothers’ incomes, educational levels, and occupational status underwent natural log transformation to reduce skewness and kurtosis. To calculate the differences/similarities in incomes, educational levels, and occupational status between parents, indices were generated by calculating the absolute difference between fathers and mothers for each SES factor. We subtracted mothers’ standardized scores from those of fathers, and absolute values were calculated to form indices. Higher and lower scores reflected greater difference and similarity between parents, respectively.

#### Coparenting

The 18-item Chinese version of the Coparenting Scale (Liu et al., 2014) was used to measure coparenting, which was the revised version based on Coparenting Scale (McHale, 1997). Mothers’ scores reflected their coparenting behavior toward fathers, and vice versa. Parents responded using a seven-point scale ranging from 1 (*absolutely never*) to 7 (*almost constantly*) to assess family integrity practices (e.g., “how often do you say or do something to invite, facilitate, or promote an affectionate or pleasant interchange between your partner and your child?”), reprimand behavior (e.g., “how often do you take a back seat while your partner deals with your child’s negative behavior?”), conflict (e.g., “how often do you argue with your partner? ”), and disparagement (e.g., “how often do you make a comment about your partner that might create negative feelings in your child?”). The Family Integrity, Reprimand, Disparagement, and Conflict subscales contain eight, three, three, and four items, respectively. Items were averaged, and higher scores reflected higher levels of family integrity practices, discipline, conflict, and disparagement. Internal consistency for the four subscales was acceptable: Family Integrity: fathers α = 0.87, mothers α = 0.86; Reprimand: fathers α = 0.61, mothers α = 0.65; Conflict: fathers α = 0.84, mothers α = 0.83; and Disparagement: fathers α = 0.89, mothers α = 0.78.

#### Paternal Involvement

Paternal involvement was measured using the 56-item Father Involvement Questionnaire (Wu et al., 2014), which is a self-report instrument consisting of three subscales: Engagement (23 items, e.g., “take my child to a museum, zoo, science center, or library”), Accessibility (eight items, e.g., “when we are not together, my child can connect with me if he/she wants to”), and Responsibility (25 items, e.g., “financial support for my child’s development”). Responses are provided on a scale ranging from 1 (*never*) to 5 (*always*), and item scores are averaged. Higher scores reflect higher levels of involvement in fathers. The internal consistency for the scale was good (α = 0.96).

#### Control Variables

Paternal involvement was influenced by child-related factors (e.g., Lundberg et al., 2007), and children’s characteristics, such as age and sex, were assessed using demographic information. These variables were treated as covariates, to ensure that their effects were controlled.

### Data Analysis

In the preliminary analysis, means and standard deviations produced for children’s characteristics, parental SES, coparenting, and paternal involvement to describe general characteristics, and correlations between these variables were calculated. Second, structural equation modeling (SEM) was performed to estimate the models for direct and indirect effects simultaneously. To calculate paternal involvement, we established latent factors prior to examining structural relationships; confirmatory factor analysis (CFA) was performed using LISREL 8.70 software (Jöreskog and Sörbom, 2004) to determine whether the latent paternal involvement construct was measured adequately by the indicators. We then produced model containing the coparenting subscales/behaviors as mediators, with children’s age and sex treated as covariates. Structural paths were tested via SEM using Mplus 7.11 software (Muthén and Muthén, 2013). The fit of the measurement and structural models was evaluated using the following fit indices (Hu and Bentler, 1999; Brown, 2006): comparative fit index (CFI; ≥0.90 acceptable, ≥0.95 excellent), Tucker–Lewis index (TLI; ≥0.90 acceptable, ≥0.95 excellent), root mean square error of approximation (RMSEA; 0.08–0.10 mediocre fit, ≤0.08 acceptable, ≤0.05 excellent), and the standardized root mean square residual (SRMR; ≤0.08 acceptable, ≤0.05 excellent). The classic goodness-of-fit index χ^2^ was also reported, but the other measures were relied upon to support the model fit (Hu and Bentler, 1999). We used a bootstrapping procedure to estimate and assess indirect effects (Preacher and Hayes, 2008). Bias-corrected confidence intervals for indirect effects were generated using 1,000 bootstrap samples, and the significance of the indirect effects was tested using 95% confidence intervals (Shrout and Bolger, 2002).

## Results

### Preliminary Analysis

Means, standard deviations, and Pearson’s correlation coefficients for children’s age and sex, SES, coparenting and paternal involvement are presented in **Table 2**. Paternal involvement was significantly correlated with all SES and coparenting behavior variables, with the exception of educational difference (*p* = 0.298), and mothers’ coparenting conflict (*p* = 0.055), in both fathers and mothers. Occupational difference was significantly correlated with family integrity practices and reprimand behavior in both fathers and mothers, but correlations between occupational difference and other coparenting measures were non-significant. All of the relationships between income and educational difference and fathers’ and mothers’ coparenting variables were non-significant, with the exception of the relationships between income difference and mothers’ reprimand behavior and educational difference and mothers’ family integrity practices. The correlations between occupational and income and educational difference were significant. The correlation coefficients for the associations between the coparenting variables varied from non-significant to moderately significant.

**Table 2:** Means, standard deviations, and correlations of covariates, SES, coparenting, and paternal involvement.

	1	2	3	4	5	6	7	8	9	10	11	12	13	14
1 Child age	1													
2 Child sex	-0.07	1												
3 Income difference	-0.08	0.15*	1											
4 Occupation difference	0.01	-0.03	0.24***	1										
5 Education difference	0.13*	-0.08	0.06	0.28***	1									
6 Father family integrity	-0.01	-0.02	-0.06	-0.17**	-0.06	1								
7 Father reprimand	0.02	-0.01	-0.08	-0.17**	-0.03	0.53***	1							
8 Father conflict	-0.06	0.06	0.04	0.04	0.10	-0.13*	-0.16*	1						
9 Father disparagement	-0.05	-0.05	0.00	0.09	-0.00	-0.11	-0.19**	0.60***	1					
10 Mother family integrity	-0.20**	0.01	-0.04	-0.17**	-0.27***	0.46***	0.25***	-0.14*	-0.06	1				
11 Mother reprimand	-0.07	-0.12	-0.16*	-0.19**	-0.11	0.32***	0.47***	-0.20**	-0.10	0.45***	1			
12 Mother conflict	0.00	0.08	0.01	-0.00	0.02	-0.06	-0.12	0.59***	0.44***	-0.13*	-0.21**	1		
13 Mother disparagement	0.02	-0.01	-0.01	0.05	0.08	-0.07	-0.13*	0.38***	0.51***	-0.13	-0.10	0.57***	1	
14 Paternal involvement	0.03	0.03	-0.13*	-0.20**	-0.07	0.69***	0.50***	-0.14*	-0.14*	0.36***	0.28***	-0.12	-0.14*	1
*M*	4.97		0.73	0.64	0.49	4.51	4.90	2.71	1.91	4.57	4.86	2.64	1.97	2.45
*SD*	1.45		0.57	0.60	0.51	1.05	1.14	1.16	1.19	1.03	1.23	1.10	1.05	0.56

M, mean; *SD*, standard deviation.

* < 0.05; ** < 0.01; *** < 0.001.

### Path Analysis of SES on Paternal Involvement

Structural equation modeling was used to test the hypotheses. We first examined the model with two latent factors of coparenting among fathers and mothers, and the model showed a bad fit. We then assessed 8-mediator including father family integrity, reprimand, conflict, and disparagement, and mother family integrity, reprimand, conflict, and disparagement while controlling for the effects of children’s age and sex. Fit statistics indicated acceptable goodness of fit for 8-mediator and CFA models, and all standardized factor loadings for latent variables were above 0.30. The goodness of fit for structural models and CFA is shown in **Table 3**.

**Table 3:** Fit statistics of structural models and CFA.

	χ^2^	*df*	*P*	CFI	TLI	RMSEA	SRMR
Structural model (2-mediator)	1483.60	110	0.00	0.72	0.62	0.14	0.10
Structural model (8-mediator)	1483.60	110	0.00	0.98	0.90	0.07	0.02
CFA (Paternal involvement)	3727.27	1481	0.00	0.95	0.95	0.08	0.07

*CFI, comparative fit index; TLI, Tucker–Lewis index; RMSEA, root mean square error of approximation; SRMR, standardized root mean square residual.*

**FIGURE 1:**
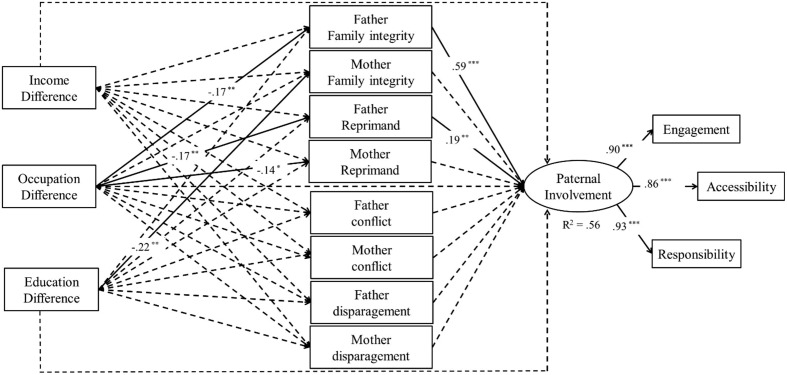
**Standardized coefficients for the SES, coparenting, and paternal involvement model; Non-significant paths are dashed; All mediators are correlated with each other; Control variables include child age and sex**.

**Figure 1** depicts the path models. Occupational difference significantly predicted fathers’ family integrity practices and reprimand behavior, and the paths between father’s family integrity and reprimand and the paternal involvement were significant. The paths between occupational difference and mothers’ reprimand behavior, and educational difference and mothers’ family integrity practices were significant. However, the paths from mothers’ family integrity practices and reprimand behavior to paternal involvement were non-significant.

We confirmed the indirect effects of SES on paternal involvement via coparenting, using bootstrapping. The indirect effects of family integrity practices (*p* < 0.01) was significant, with 95% bootstrapping confidence intervals for the mediating effects ranging from -0.16 to -0.03. Other indirect effects were non-significant, with confidence intervals ranging between -0.09 and 0.07. Therefore, fathers’ family integrity mediated the relationship between occupational difference and paternal involvement. We further estimated the indirect effect was -0.10.

## Discussion

The main finding of the present study was that occupational difference influenced paternal involvement through fathers’ family integrity practices. Income and educational difference was not associated with paternal involvement, either indirectly, via coparenting, or directly. The results partly supported the hypothesis that difference between paternal and maternal SES would predict paternal involvement. To our knowledge, this study was among the first to use difference between fathers’ and mothers’ individual incomes, occupations, and educational levels to elucidate the influence of SES on paternal involvement. In the past two decades, popular interest in research examining fathering has increased in China (Shwalb et al., 2010). This study extends the results of previous research involving Chinese fathers by considering fathers’ engagement, accessibility, and responsibility, which capture the overall circumstances surrounding father–child interactions (Lamb and Tamis-Lemonda, 2004).

The results of the analysis showed that difference between fathers’ and mothers’ occupational status exerted negative influence on paternal involvement. This result supported the notion that the gender gap in employment results in deficiencies in paternal involvement (Shwalb et al., 2010). Relative to fathers, mothers are more likely to hold less demanding jobs and choose family friendly occupations (Raley et al., 2012), which could lead to a difference in occupational status between fathers and mothers. According to the percentages representing occupational status in **Table 1**, mothers’ occupational status was generally lower relative to that of fathers. We speculated that mothers with lower occupational status were less powerful within the family relative to fathers with higher occupational status. Some involvement activities, such as taking children to the doctor, are more onerous, and powerful fathers may have unloaded these tasks onto mothers (Raley et al., 2012), which supports the relative resource theory. In addition, similarities between fathers’ and mothers’ occupational status could facilitate paternal involvement. When both parents’ occupational status was high or low, fathers were more likely to become involved in childrearing relative to fathers whose occupational status was higher than that of their spouses. The results concerning parental resource discrepancies between fathers and mothers extended those of previous research (e.g., Deutsch et al., 1993). We used absolute difference values to form the indices, which allowed us to examine the extent of differences.

The results also indicated that SES indicators were outcome specific (Conger and Donnellan, 2007). Occupational, rather than income or educational, difference influenced paternal involvement. Occupational difference was correlated with both income and educational difference. We noted that occupational difference predicted paternal involvement after controlling for income and educational difference. The results suggested that social capital (Conger and Donnellan, 2007) plays an important role in the Chinese family dynamic. It is possible that the traditional Chinese view that “men are chiefly responsible for activity in society while women are responsible for the home” (Shwalb et al., 2010) has faded because of increased labor force participation in mothers. We speculated that the importance of the mother’s social role within the family could have increased. Increases in mothers’ social capital could facilitate fathers’ participation in childrearing. Our findings showed that similarities between fathers’ and mothers’ social capital contributed to paternal involvement.

The systemic context framework can be used to understand fathering, indicating that coparenting is the principal intrafamilial determinant of paternal involvement (Doherty et al., 1998). In the present study, we explored the mediating role of coparenting in the association between SES and paternal involvement. Consistent with our hypothesis, the results showed that difference in occupational status between fathers’ and mothers’ could hinder paternal involvement by reducing fathers’ family integrity practices. The results also supported the ecological model of coparenting (Feinberg, 2003), which suggested that coparenting mediated the relationship between socioeconomic factors and paternal involvement.

Family integrity practices reflected fathers’ attempts to promote a sense of togetherness between family members (McHale, 1997). The results suggested that occupational, rather than income or educational, difference played an import role in fathers’ attempts to improve togetherness. Further, consistent with the results of previous studies (e.g., Morrill et al., 2010; Goldberg, 2015), the results indicated an association between coparenting behavior and paternal involvement. The mediating role of coparenting also supported the notion of the family system (Minuchin, 1985). The results suggested that a difference in individual characteristics between parents influenced the father–child subsystem via the parental subsystem.

The findings of the present study should be viewed in light of several limitations. First, the cross-sectional nature of the data made it impossible to determine the long-term influence of SES on paternal involvement. Longitudinal research could clarify the strength of the relationships between SES and parenting practices. Second, although internal consistency was generally acceptable, low internal consistency for the Reprimand subscale (fathers α = 0.60, mothers α = 0.65) could have compromised the results. Third, self-report of paternal involvement and coparenting may influence the study’s conclusions. Fourth, the latent model indicated a bad fit, and the 8-mediator model could have compromised the test of mediating role of coparenting. In addition, the generalization of the results was limited by the sample. Generalization concerning Chinese fathers was difficult because of within-culture variations in fathering (Shwalb et al., 2010). China includes 56 major ethnic groups, and the present sample was not entirely representative of the national population. Further research should focus on comparisons of Chinese paternal involvement between ethnic groups, which would improve understanding of the true picture of fathering in China.

Notwithstanding these limitations, the study used differences/similarities between paternal and maternal SES to explore parents’ contributions and increased our understanding of the associations between SES and paternal involvement, via coparenting. Importantly, SES indicators appeared outcome specific with respect to paternal involvement, and fathers’, rather than mothers’ coparenting, played a mediating role. Our findings could have important practical implications. Parenting programs should focus on parents with greater occupational differences, and these families should be considered high risk in clinical interventions. Teaching fathers to express their emotions and practice positive behaviors to improve family togetherness could be helpful.

## Conclusion

Our findings indicated that SES indicator measures were outcome specific, and occupational differences/similarities exerted an influence on paternal involvement indirectly, via coparenting.

## Author Contributions

Conception and design of the study: CL, XW. Acquisition, analysis, and interpretation of data: CL, XW, SZ. Drafting the work and revising it critically for important intellectual content: CL, XW, SZ.

## Conflict of Interest Statement

The authors declare that the research was conducted in the absence of any commercial or financial relationships that could be construed as a potential conflict of interest.
